# Molecular Analysis of rs2070744 and rs1799983 Polymorphisms of NOS3 Gene in Iranian Patients With Multiple Sclerosis

**DOI:** 10.18869/nirp.bcn.8.4.279

**Published:** 2017

**Authors:** Mohammad Mehdi Heidari, Mehri Khatami, Yaser Tahamtan

**Affiliations:** 1. Department of Biology, Faculty of Science, Yazd University, Yazd, Iran.; 2. Department of Biology, Ashkezar Branch, Islamic Azad University, Yazd, Iran.

**Keywords:** Multiple sclerosis, NOS3 gene, rs2070744, rs1799983, Polymorphism

## Abstract

**Introduction:**

Multiple Sclerosis (MS) is a disease of central nervous system that mainly causes lesions or plaques in the spinal cord and brain. The purpose of this study was to analyze the relation between c.-813C>T (rs2070744) and c.894G>T (rs1799983) polymorphisms of NOS3 gene and MS in Iranian patients.

**Methods:**

A total of 78 patients with MS and 80 healthy controls were screened for NOS3 (rs2070744 and rs1799983) Single Nucleotide Polymorphisms (SNPs) by tetra-primer multiplex ARMS-PCR and PCR-RFLP.

**Results:**

Genotype frequencies of the c.-813C>T polymorphism in patients compared to controls were as follows: 53.8% to 80.0% for TT genotype, 41.0% to 18.8% for TC genotype, and 5.1% versus 1.2% for CC genotype (P=0.001). The frequencies of GG genotype was 57.7% and 78.8% and for GT genotype of c.894G>T polymorphism in patients compared to control subjects was 42.3% and 21.2%, respectively (P=0.004).

**Conclusion:**

Our results indicate that the studied NOS3 polymorphisms may be associated with MS in Iranian patients.

## 1. Introduction

Multiple Sclerosis (MS) is a neuroimmunological disease, causing severe neurological disabilities as a result of demyelination (Goris, Pauwels, & Dubois, 2012). It is mainly defined as a heterogeneous, complex, and multifactorial disease. The disease becomes progressive after several episodes in the majority of affected individuals (Hoffjan & Akkad, 2010; McElroy & Oksenberg, 2011). Although twin and sibling pair studies point to a genetic component, the etiology of MS is mostly unknown. However, there is a seminal effort to detect the responsible genes in increased susceptibility and affecting clinical consequence (Hoffjan & Akkad, 2010).

An important role for Nitric Oxide (NO) in the pathogenesis of MS and its influence on the various aspects of the disorder, including changes in synaptic transmission, inflammation, and neuronal death were pointed by abundant evidence (Gerzanich, Ivanova, Zhou, & Simard, 2003; Jagnandan, Sessa, & Fulton, 2005). Several studies are shown that nitrate, NO degradation product, is found with increased concentration in cerebrospinal fluid of patients with MS (Giovannoni, 1998; Tajouri et al., 2004).

Oxidation of L-arginine to Nitric Oxide (NO) is catalyzed by Nitric Oxide Synthases (NOSs). An isoform of NO producing enzymes is endothelial Nitric Oxide Synthase (NOS3) that is constitutively expressed in endothelial cells. This enzyme has been found to play a prominent role in both vasculogenesis and angiogenesis. The nitric oxide synthases is encoded by NOS3 gene located on chromosome 7q35-q36 (Forstermann & Sessa, 2011). There are two common polymorphisms of NOS3 gene in many populations that are associated with NOS3 enzyme activity and production. In the seventh exon of the NOS3 gene, the c.894G>T SNP substitutes glutamic acid at codon 298 to aspartic acid (Glu298Asp).

This polymorphism causes diminished NOS3 enzyme activity and the c.-813C>T (rs2070744) polymorphism in the promoter region, where it has been found with reduced NOS3 promoter activity in vitro (Miyamoto et al., 1998; Veldman et al., 2002). The genetic association of these NOS3 gene polymorphisms and susceptibility to MS have identified in several studies (AlFadhli, Mohammed & Al Shubaili, 2013; Blanco, Yague, Graus, & Saiz, 2003; Modin et al., 2001; Tajouri et al., 2004). The purpose of the present research was to determine a relation between c.-813C>T (rs2070744) and c.894G>T (rs1799983) polymorphisms of NOS3 gene with susceptibility to MS in Iranian population.

## 2. Methods

### 2.1. Study subjects

After a neurological examination, 78 patients with MS (60 females and 18 males) and the mean age of 33.74 years (age range from 24–57 years) were recruited from MS Clinic (Yazd, Iran). Healthy volunteers (80 subjects) were randomly selected from their families with no history of autoimmune and neurological diseases. In the present study, the experimental and control groups were ethnically matched. All individuals gave informed consent for DNA test. DNA extraction was performed from whole blood with salting out method according to the manufacturer’s instructions. Patients and controls’ blood and DNA samples were isolated and stored at −20°C.

### 2.2. Genotyping of NOS3 (rs2070744) by Multiplex-ARMS PCR

Genotyping of c.-813C>T NOS3 SNP (rs2070744) was detected by tetra-primer ARMS-PCR (T-ARMS-PCR). PCR amplification of c.-813C>T polymorphism in the 5′ flanking region of the NOS3 gene was performed using four primers. The online website Primer1 was used for design of our primers (Table 1). The primers specificity were tested with ‘BLAST’ program in NCBI server. To improve the specificity of T-ARMS-PCR, the 3rd nucleotide from the 3′-terminus of inner primers was changed to destabilize mismatch.

**Table 1:** PCR primers and conditions.

**eNOS Gene**	* Primer Sequence	**Temperature**	**Amplicon Size**
SNP ID: rs2070744	F1: 5′-CTACAAACCCCAGCATGCACTC-3′	66°C	T allele (150bp) C allele (211bp) Control band (319bp)
	R1: 5′-CATTAGGGTATCCCTTCCCCTC-3′	66°C	
	F2: 5′-GGGCATCAAGCTCTTCCCTGTCC-3′	72°C	
	R2: 5′-TAGGGCTGAGGCAGGGTCAGACA-3′	70°C	
SNP ID: rs1799983	F3: 5′-TCACGGAGACCCAGCCAATGAG-3′	70°C	G allele Undigested with MboI (293bp)
	R3: 5′-TCCATCCCACCCAGTCAATCCC-3′	70°C	T allele Digest with MboI (197+95bp)

* F1, F2, R1 and R2 primers designed for T-ARMS-PCR and F3 and R3 for PCR-RFLP reaction.

**Figure 1:**
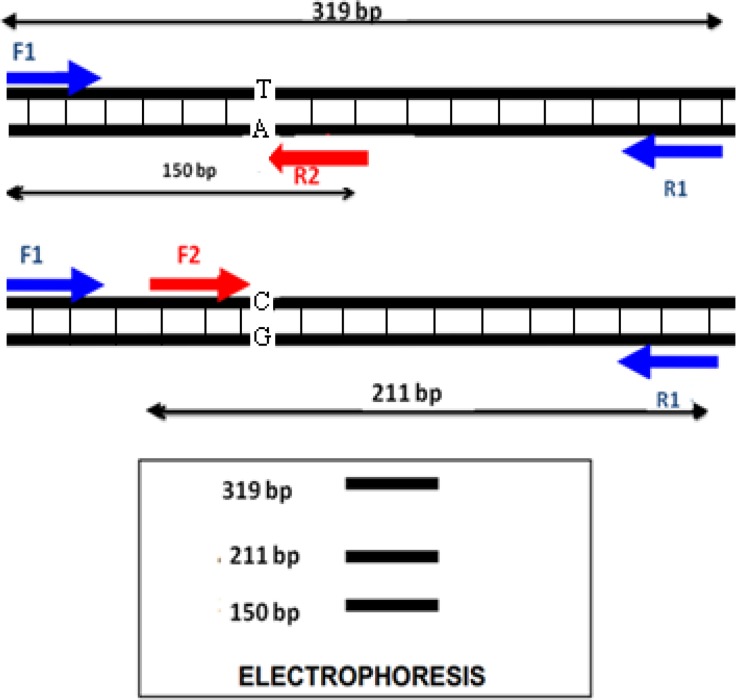
Schematic presentation of T-ARMA-PCR technique for c.-813C>T polymorphism detection. A larger (non-allele-specific) control amplicon and two different allele-specific amplicons are generated by a pair of two common (outer) primers and two allele specific (inner) primers.

**Figure 2:**
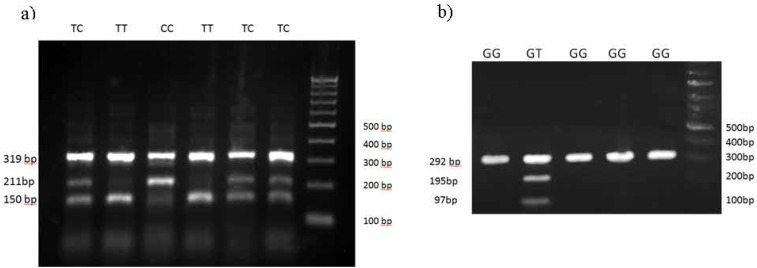
a) Results of T-ARMS-PCR of rs2070744 polymorphism and b) PCR-RFLP of rs1799983 by MboI restriction endonuclease.

PCR was achieved in 25 μL volume, containing 50 ng of DNA, 1X PCR Master Mix, and 250 nM of each primer (Yekta Tajhiz Azma, Tehran, Iran). Touchdown PCR was performed at 95°C for 2 min, denaturation at 95°C for 20 s, the first 10 cycles annealing temperature at 68°C to 60°C and remaining 25 cycles at annealing at 59°C for 60 s and extension at 72°C for 50 s. The PCR products were run on 2% agarose gel electrophoresis. In this method, the gel electrophoresis revealed 3 bands in AG heterozygotes (319, 150, and 211bp), 2 bands in AA homozygotes (319 and 150 bp), and 2 bands in GG homozygotes (319 and 211 bp) (Figures 1 and 2).

### 2.3. Genotyping NOS3 (rs1799983) by PCR-RFLP

The NOS3 polymorphism c.894G>T introduces the restriction site for MboI endonuclease. PCR was achieved in 25 μL volume; containing 50 ng of DNA, 1X PCR Master Mix, and 250 nM of each primer (Yekta Tajhiz Azma, Tehran, Iran). The PCR reaction was according to the following stages: 35 cycles; including denaturation, annealing, and extension at 95°C, 64°C, and 72°C for 35 s, respectively; and a final extension at 72°C for 5 min. According to Figure 2, a 292 bp fragment was detected in GG homozygote genotypes (MboI-undigested), also 197 and 95 bp fragments in TT homozygote genotypes (MboI-digested), finally 292, 197, and 95 bp fragments in GT heterozygote genotypes (MboI-digested and undigested fragments).

### 2.4. Statistical analysis

Statistical analysis was performed using SPSS (IBM SPSS 22, SPSS Inc., Chicago, IL., USA). Allelic and genotypic frequencies were calculated for each SNPs. Multiple logistic regression models (co-dominant, dominant, recessive, and over-dominant) were employed to analyze the genetic data. P value less than 0.05 was considered statistically significant.

## 3. Results

The genotype distributions and allele frequencies of two types of polymorphism in NOS3 gene, c.-813C>T, and c.894G>T were analyzed in Iranian patients with MS and healthy controls. Table 2 presents genotypic and allelic frequencies of c.-813C>T polymorphism among the patients and controls group. In the patients group, the frequencies of TT, CT and CC genotypes were 53.84%, 41.02%, and 5.1%, respectively. In the control group, the frequencies of TT, CT, and CC genotypes were 80.0%, 18.8% and 1.2%, respectively. Statistical analysis showed that c.-813C>T polymorphism is associated with risk of MS according to co-dominant, dominant, and over-dominant model (co-dominant model, P=0.001; dominant model, P=0.004; and over-dominant, P=0.002). The prevalence of C allele was significantly higher in patients compared to the control group (OR: 0.345, 95%CI: 0.18–0.64; P=0.001).

**Table 2:** Genotype counts and allele frequencies c.-813C>T (rs2070744) polymorphism in patients and controls.

	**Genotype/Allele**	**MS Patients (n=78)**	**Healthy Controls (n=80)**	**Odds Ratio (95% CI)**	**P**
Co-dominant model	TT	42(53.8%)	64(80.0%)	1(ref.)	0.001
	TC	32(41.0%)	15(18.8%)	3.25(1.57–6.72)	
	CC	4(5.1%)	1(1.2%)	6.10(0.66–56.44)	
Dominant model	TT	42(53.8%)	64(80.0%)	3.43(1.69–6.95)	0.004
	TC+CC	36(46.2%)	16(20.0%)		
Recessive model	TT+TC	74(94.9%)	79(98.8%)	4.27(0.47–39.09)	0.150
	CC	4(5.1%)	1(1.2%)		
Over-dominant model	TT+CC	46(59.0%)	65(81.2%)	3.01(1.47–6.19)	0.002
	TC	32(41.0%)	15(18.8%)		
Allele frequency	T	116(74.4%)	143(89.4%)	0.345(0.18–0.64)	0.001
	C	40(25.6%)	17(10.6%)		

Genotypic and allelic frequencies of c.894G>T polymorphism among the patients and controls group were shown in Table 3. In the patients group, the frequencies of GG, GT, and TT genotypes were 57.7%, 42.3%, and 0.0%, respectively. In the control group, the frequencies of GG, GT, and TT genotypes were 78.7%, 21.2%, and 0.0%, respectively. Statistical analysis showed that according to co-dominant model, c.894G>T polymorphism is associated with risk of MS (P=0.004). The prevalence of T allele was significantly higher in patients compared to the control group (OR: 0.443, 95% CI: 0.23–0.83; P=0.013).

**Table 3:** Genotype counts and allele frequencies c.894G>T SNP (rs1799983) in patients and controls.

**Genotype/Allele**	**MS Patients (n=78)**	**Healthy Controls (n=80)**	**Odds Ratio (95% CI)**	**P**
Co-dominant model	45(57.7%)	63(78.8%)	1(ref.)	
GG				0.004
GT	33(42.3%)	17(21.2%)	2.72(1.35–5.47)	
TT	0(0.0%)	0(0.0%)		
Allele frequency				
G	123(87.8%)	143(89.4%)		
T	33(21.2%)	17(10.6%)	0.443(0.23–0.83)	0.013

## 4. Discussion

The involvement of Nitric Oxide (NO) in MS had been suggested after the discovery of the critical role of NO in inflammation. However, due to the opposing roles of NO in cellular processes, the extent of NO contribution to MS is not yet fully understood. In this study, we found significant differences in the distribution of c.-813C>T and c.894G>T polymorphisms of the NOS3 gene in Iranian patients with MS compared with healthy population. Several reports have been showed that c.-813C>T (rs2070744) polymorphism in the 5′ flanking (promoter) region of the NOS3 gene is associated with coronary spasm and vascular disease (Casas et al., 2006; Kajiyama et al., 2000; Nakayama et al., 1999; Nakayama et al., 2000). Also, previous studies have demonstrated the association between this polymorphism and recurrent abortions and male infertility (Luo et al., 2013; Safarinejad, Shafiei, & Safarinejad, 2010). This SNP significantly reduces NOS3 promoter transcription activity, inhibiting NO production which ends in endothelial dysfunction. The present study demonstrated that this polymorphism was associated with the MS in Iranian population and might play an important role in our patients. The frequency of C allele in our patients was significantly higher than control subjects (P=0.001). To our knowledge, the current research is the first to study the correlation of rs2070744 with MS. The physiological and pathological effects of c.-813C>T polymorphism, however, requires further research.

The present study is the first to reveal an association between the c.894G>T SNP (rs1799983) of the NOS3 (Glu298Asp variant) gene and susceptibility to MS (P=0.004). We aimed to elucidate the missense Glu-298Asp polymorphism within exon 7 of the NOS3 gene in 78 Iranian MS patients and 80 healthy controls. Some authors have suggested that Glu298Asp polymorphism in NOS3 gene plays a role in various diseases such as idiopathic male infertility, myocardial infraction, and placental abruption (Gad et al., 2012; Luo, Wen, Zhou, Chen, & Zhang, 2014; Safarinejad et al., 2010; Yoshimura, Yoshimura, Tabata, Yasue, & Okamura, 2001). The c.894G>T SNP alters the stability, biological half-life, and activity of the enzyme and is associated with reduced NO production (Wang et al., 1997).

Several findings have revealed that this mutation was the result of a conformational change in the eNOS protein from helix form to tight turn (Joshi, Mineo, Shaul, & Bauer, 2007). Based on our expectation, the statistical difference in this SNP indicates the correlation of G allele of rs1799983 with MS patients compared with controls. Thus, G allele had the greatest effects in Iranian patients. Our results show that NOS3 polymorphisms may be associated with MS in Iranian patients and so these variants might influence the risk of MS, specifically in the Iranian population.
